# Pediatric biobanks and parents of disabled children associations opinions on establishing children repositories in developing countries

**DOI:** 10.25122/jml-2020-0106

**Published:** 2021

**Authors:** Svetlana Mykolaivna Gramatiuk, Irina Yuriivna Bagmut, Michael Ivanivich Sheremet, Karine Sargsyan, Alla Mironovna Yushko, Serhii Mykolaevich Filipchenko, Vitaliy Vasilyevich Maksymyuk, Volodimir Volodimirovich Tarabanchuk, Petro Vasilyevich Moroz, Andriy Ivanovich Popovich

**Affiliations:** 1. Ukraine Association of Biobank, Institute of Cellular Biorehabilitation, Kharkiv, Ukraine; 2. Kharkiv Medical Academy of Postgraduate Education, Kharkiv, Ukraine; 3. Surgical department No.1, Bukovinian State Medical University, Chernivtsi Ukraine; 4. Biobank Graz, Medical University of Graz, Graz, Austria; 5. Yaroslav Mudryi National Law University, Kharkiv, Ukraine; 6. Kharkiv Medical Academy of Postgraduate Education, Kharkiv, Ukraine; 7. Department of Pathology (Pathology and Forensic Medicine), Bukovinian State Medical University, Chernivtsi, Ukraine

**Keywords:** pediatric biobanking, specimens of sick children, ethical and legal aspects

## Abstract

Pediatric biobanks are an indispensable resource for the research needed to bring advances in personalized medicine into pediatric medical care. It is unclear how or when these advances in medical care may reach children, but it is unlikely that research in adults will be adequate. We conducted the screening for a hypothetic problem in various European and American pediatric biobanks based on online surveys through e-mail distribution based on the Biobank Economic Modeling Tool (BEMT) questionnaire model. Participants in the survey had work experience in biobanking for at least 3 years or more. Contact information about the survey participants was confirmed on the social networks profiles (LinkedIn), as well as on generally available websites. First, we tried creating a model which can show the pediatric preclinical and basic clinical phase relationship and demonstrate how pediatric biobanking is linked to this process. Furthermore, we tried to look for new trends, and the final goal is to put the acquired knowledge into practice, so medical experts and patients could gain usable benefit from it. We concluded that leading positions must take into account ethical and legal aspects when considering the decision to include children in the biobank collection. However, communication with parents and children is essential. The biobank characteristics influence the biobank's motives to include children in the consent procedure. Moreover, the motives to include children influence how the children are involved in the consent procedure and the extent to which children are able to make voluntary decisions as part of the consent procedure.

## Introduction

The role of biobanks in biological research in general and their impact on medical, societal and economic issues have been discussed in two reports from the Organization for Economic Co-operation and Development. In biomedical research, irreproducible results are increasingly recognized as a major threat for scientists and the public and causing significant losses of private and public investments in research. Recent investigations have revealed that unreliable results are caused to a large extent by poor biological reagents and reference materials [1–4].

Children's biobanks are a new direction, and the level of protection in research involving minors needs to be considered as outlined in the 2010 Secretary's Advisory Committee, Provincial Health Authority. The level and precautions used in clinical trials involving minors (EU Regulation No. 536/2014) differ significantly when examining pediatric biological material in a biobank, which must be taken into account when creating a pediatric repository [5, 6].

The progress of medical and pharmaceutical research in the world, as well as in Ukraine, directly depends on the quality of human bio-samples. 

Pediatric biobanks are an indispensable resource for the research needed to bring advances in personalized medicine into pediatric medical care. It is not yet clear how or when these advances in medical care may reach children, but it is unlikely that research in adults will be adequate [5 – 9, 10, 11].

Today, more than 120 biobanks are known in the world, and at least three global associations and networks are the Middle Eastern and African Society for Biobanking (ESBB), International Society for Biological and Environmental Repositories (ISBER), and Biobanking and BioMolecular Resources Research Infrastructure (BBMRI). However, only 20% of them have biological collections related to pediatrics.

For the practice of personalized medicine to be applied to children, genomic research will need to be conducted in children [10–16]. An economical and efficient approach to such research is to develop genomic biobanks using biological samples and health information collected from children.

In this study, we explored the opinions and attitudes of biobanks and parents' associations towards the donation of specimens from sick children to a hypothetical biobank.

## Material and Methods

For the tool development, a survey was designed and sent to 230 biobank managers and directors worldwide. We have created a list of about 90 managers, directors in biobanking active individual researchers in developing countries with considerations to ensure wide geographical coverage. The focus was on the active sides for the most accurate evaluation of the survey data. The estimated percentage of respondents was 20–25%.

In order to achieve our goals, we conducted a multidisciplinary review of the pediatric biotechnology market to refine and collect information on economic models. We conducted the screening for a hypothetic problem in various European and American pediatric biobanks based on online surveys through e-mail distribution based on the Biobank Economic Modeling Tool (BEMT) questionnaire model [17].

The survey with detailed questions has been divided into five sections:

• biobank demographics and structure;
• ethical aspects of pediatric biobank;
• legal aspects of pediatric biobank;
• question of communication biobank-personal/physician and parents’ associations towards the donation of a specimen from sick children;
• samples quality control.

The managers, directors, researchers of biobanks, and parents of disabled children associations were identified using social networks (LinkedIn), as well as generally available websites. Participants in the survey had work experience in biobanking for at least 3 years or more. The request for a contribution to the survey has been spread by means of blind e-mail, and no personal data of a participant was available to others at this stage. The e-mail comprised a short invitation letter and info about the survey with an attached link to the survey. Responders had the opportunity to save and exit the survey several times and had 4–6 weeks for completion of the survey submission altogether. Three follow-up e-mail reminders were sent to all participants in two-week frequencies. 

Data were collected from several sources in each biobank after obtaining the permission of officials (managers): websites, information on paper (informed consent forms) that characterize biobanks, and consent policies. Analysis of the collected material made it possible to develop information letters for employees of biobanks, parents, and children. The main direction was the selection of people with sufficient experience in building a children’s protocol and consent procedures in this direction. However, the work experience showed that there was not enough to attract specialists. Therefore, the participating children and parents were also involved [18–21, 22]. Data on general management and possible implementation in biobanks was requested. Following data sources for identifying survey answers were applicable: data from management and governance documents, team meeting protocols (with other responsible staff), strategic concept, defined activities, data on the quality management system, and strategy matrix. After collecting survey replies, interviews have been stated to precise the information and marge the possible covered market.

## Results

We have received only approximately 23% responses; 56 biobanks have responded, and the diversity of biobanks gave an extended profile. For example, in one subgroup, there were differences in operation directions - from collections of one disorder or organ to collections of almost all possible biological samples of human nature in biobanks of medical centers. A total of 56 responses were received. Of the 56 survey responses received, 32 were complete, and 24 were partially complete. Managers, directors, and researchers of pediatric biobanks typically completed the ﬁrst two sections (i.e., biobank demographics and structure, ethical aspects of pediatric biobanks). In contrast, associations of parents of disabled children completed the sections concerned with the legal aspects of the pediatric biobank, questions on the communication between the biobank and individual/physician, and the donation of a specimen from sick children). 

**Figure 1.**
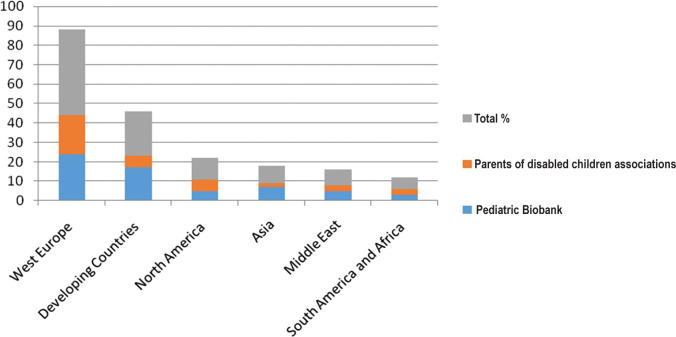
Demographics of survey respondents.

The majority of the survey responses were from West Europe (44%), developing countries (23%), and North America (11%), with the remaining surveys from Asia, Middle East, South America, and Africa (Figure 1).

**Figure 2.**
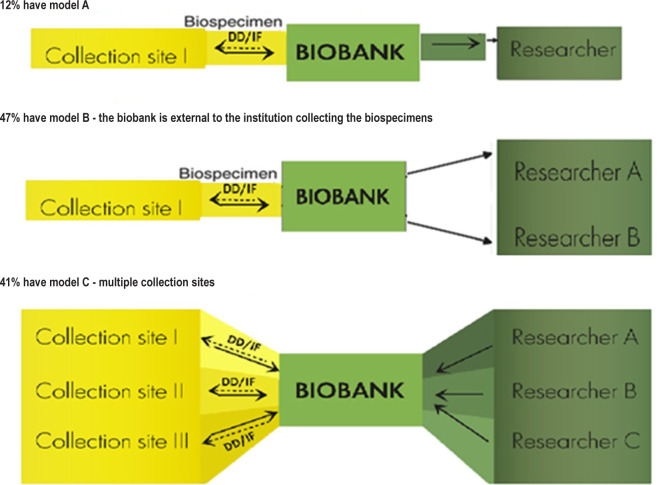
Model biobank demographics and structure. DD – diagnostic discrepancy; IF – incidental finding.

We found that 12% of biobanks have the A structure model - the collection site and the biobank reside within the same institution, 47% have model B - the biobank is external to the institution collecting the biospecimens and 41% have model C - multiple collection sites (Figure 2). Solid arrows represent the transfer of bio-specimens, and dashed arrows represent a diagnostic discrepancy (DD) or incidental finding (IF).

### Ethical and legal aspects of a pediatric biobank

Based on the survey results, we found that there are no specific regulations in many developing countries to control the creation and management of pediatric biobanks arising from institutional research projects. Also, major ethical concerns need to be met. These reasons are often left to be managed by the local Ethical Committees and their affiliated institution. As a result of the questionnaire, in developing countries, 76% of pediatric biobanks do not have consent forms of exceptional permission for specimen collection.

An important component for the management of the pediatric biobank is the process of counseling minors; it is the involvement of children in this issue that is the basis for the management and sustainable development of the pediatric biobank.

### A question of communication

As a result of the analysis of the respondents’ answers, we established that personal communication and trust are the main factors influencing the donation of samples to the pediatric biobank.

The earned trust, and the child’s personalized consent can lead to constant interaction, stability in the research infrastructure, and an increase in feedback among its participants, which is based on respect for minors. Therefore, it is a matter of communication to obtain a child's consent.

According to the results of our survey, we developed suggestions for establishing and maintaining communication with children and adolescents:

1. Establishing rapport;
2. Maintaining rapport;
3. Keeping the child in the conversation;
4. Maintaining a supportive environment;
5. Providing information in an accessible format for the child (cartoon, fairy tale, video game).

### Risk management and pediatric biobanking

Long-term planning with the inclusion of a full range of risks requires working with cell lines, products containing DNA/RNA and biologically active substances.

**Figure 3.**
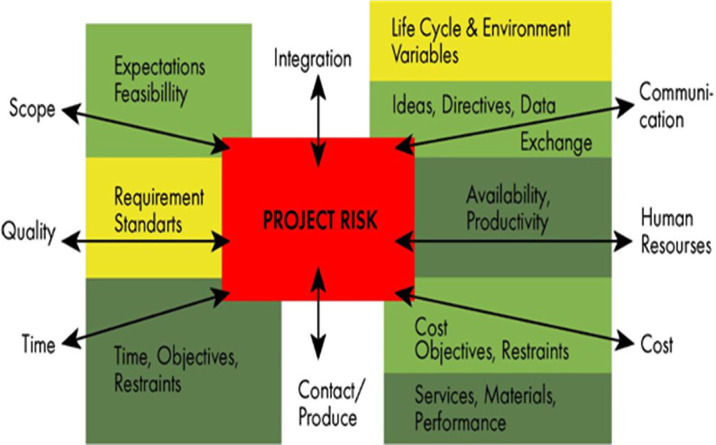
Risk management and pediatric biobanking.

The division into categories when working in a pediatric biobank is as follows: reputation-related risks, ethical risks, financial risks, operative risks, standard lab risks, human resources (HR) risks, infrastructural risks, information technology (IT) risks, strategic risks, natural disasters (Figure 3).

Based on the fact that the identification and assessment of risks is a subjective component, we recommend a risk analysis at a seminar with the participation of all employees of the pediatric biobank such as management staff, public relations (PR), HR, financial officers, lab technicians, researchers, IT specialists, ethics specialists, infrastructure specialists, medical doctors and carrier organization members (as strategy makers and stakeholders).

We revealed potential risks for pediatric biobanking in developing countries:

1. Children and some parents demonstrated a desire for re-consent and the right to withdraw at age 18;
2. Parents often wanted the option of receiving their children’s genetic results, though the return of results was seen as potentially infringing on the privacy and autonomy of the child;
3. Despite the rising social expectation that research participants’ voices be heard and an increasing need for pediatric biobanks, there is a paucity of empirical research conducted to date.

## Discussion

According to the results of our survey, the main models of the pediatric biobank were model A, where the collection site and the biobank reside within the same institution. In model B, the biobank is external to the institution collecting the biospecimens, but researchers may be internal or external to the collecting institution. In model C, multiple collection sites contribute with biospecimens to a centralized biobank, the biobanking network, which distributes biospecimens to researchers [22–25]. The decision of which model to choose for a pediatric biobank in developing countries remains to be decided by the organizer and sponsor.

Interestingly, there was a complete lack of understanding of the pediatric biobanks’ structure in developing countries. There were no regulatory documents necessary for its creation. Scientific, medical and pharmaceutical institutes and clinics have collected, processed and stored biological material without standard operating procedures. Such kind of situation led to a massive number of mistakes in various scientific departments, which caused the reduction of the competitiveness of medical science in developing countries in general [26–27].

Another problem area of the biobanks network in developing countries is connected with the resellers who were involved in the resale of services related to the biological material. They are mainly focused on the movement of tissues and biological samples from primary sources into research groups. The employees of such companies are almost entirely made of people without medical education. This situation led to a greater volume of mistakes. 

We found that biobanks make “one-time efforts,” namely a one-time collection, only for a specific purpose, as well as in the absence of a clear research motive, and this emphasizes the importance of showing respect for the child, which should be the main goal of biobanks since research interests go hand in hand with the interests of the child. This is the driving force behind the European Pediatric Translational Research Infrastructure (EPTRI), which is a strong incentive to involve children in the consent process [28].

Scientific and medical research that includes children is essential to developing therapies for younger patients [29]. Pediatric biobanking using samples from minors provides a critical and expanding resource for health-related research. 

Undoubtedly, ethical and legal issues based on consultations with stakeholders emphasize the importance of an appropriate framework for practice. The actual parental consent was considered by many to be sufficient, while the undoubted importance of children’s consent for research in the field of biobanks was not studied.

The development of biobanks for children requires a deep understanding of many ethical, legal and social issues. This is the solution to the missing sections, and the elimination of gaps at the legislative level, as an example of the lack of attention to the rights of vulnerable subjects in some developing countries, should be considered [30].

## Conclusion

We concluded that leading positions must take into account ethical and legal aspects when considering the decision to include children in the biobank collection. However, communication with parents and children is essential. The procedure for including minors in the consent procedure is significantly influenced by the characteristics and purpose of the pediatric biobank. This is what affects the degree of participation in the consent procedure for children. Our study confirms the need for specific policies dedicated to pediatric biobanks by highlighting how the nature of the disease affecting children may influence the parents’ opinions and decisions towards the enrolment of their children in biobank-based research studies.

## Acknowledgments

### Conflict of interest

The authors declare that there is no conflict of interest.
